# α-Hydroxybutyrate dehydrogenase is associated with atherothrombotic events following infrainguinal angioplasty and stenting

**DOI:** 10.1038/s41598-019-54899-0

**Published:** 2019-12-03

**Authors:** Silvia Lee, Renate Koppensteiner, Christoph W. Kopp, Thomas Gremmel

**Affiliations:** 1 Department of Internal Medicine II, Medical University of Vienna, Vienna, Austria; 2 Department of Internal Medicine, Cardiology and Nephrology, Landesklinikum Wiener Neustadt, Wiener Neustadt, Austria

**Keywords:** Peripheral vascular disease, Restenosis

## Abstract

Besides clinical characteristics, easy-accessible laboratory markers could be of value to refine risk stratification in peripheral artery disease. In the current study, we investigated whether α-hydroxybutyrate dehydrogenase (HBDH) is associated with atherothrombotic events in 83 stable patients undergoing infrainguinal angioplasty and stenting. The primary endpoint was defined as the composite of the first occurrence of nonfatal myocardial infarction, nonfatal stroke or transient ischemic attack and cardiovascular death within 2 years after angioplasty and stenting, and occurred in 6 patients (7.2%). HBDH levels at baseline were significantly higher in patients who subsequently developed the primary endpoint (126 U/L [116–137 U/L] vs. 105 U/L [95–120 U/L]; p = 0.04). ROC curve analysis revealed that HBDH could distinguish between patients without and with future atherothrombotic events. A HBDH concentration ≥ 115 U/L was identified as the best threshold to predict the composite endpoint, providing a sensitivity of 83.3% and a specificity of 71.4%, and was therefore defined as high HBDH. High HBDH was seen in 28 patients (33.7%). Ischemic events occurred significantly more often in patients with high HBDH than in patients with lower HBDH levels (5 vs. 1 patients, p = 0.007). In conclusion, HBDH is associated with the occurrence of atherothrombotic events after infrainguinal angioplasty with stent implantation. Future trials are warranted to study the predictive role of HBDH for ischemic outcomes and to investigate underlying mechanisms.

## Introduction

Patients with peripheral artery disease (PAD) are at an increased risk of atherothrombotic events like myocardial infarction and stroke^1^. Moreover, previous studies have shown that those undergoing angioplasty and stenting for PAD frequently suffer target vessel restenosis or reocclusion^2–4^. While the latter are predominantly a consequence of intimal hyperplasia^5^, atherothrombotic events are often initiated by plaque rupture with subsequent platelet and coagulation activation^6^, and may therefore be prevented by more intense antithrombotic therapy^7,8^. Indeed, a new antithrombotic regimen consisting of platelet inhibition with low-dose aspirin and inhibition of thrombin generation with a very low dose of the factor Xa antagonist rivaroxaban recently yielded promising results in patients with stable PAD^9^. In detail, rivaroxaban 2.5 mg twice daily in additon to 100 mg aspirin significantly reduced the occurrence of atherothrombotic events compared to 100 mg aspirin alone in 7470 PAD patients of the COMPASS (Cardiovascular Outcomes for People Using Anticoagulation Strategies) trial. However, the decrease in ischemic outcomes was achieved at the cost of an increased major bleeding risk^9^. Consequently, the new treatment regimen should only be prescribed in PAD patients at high risk of atherothrombotic events. In order to optimally select PAD patients in need of intensified antithrombotic therapy, it seems crucial to identify factors associated with adverse ischemic outcomes. Besides clinical characteristics, easy-accessible laboratory markers could be of value to refine risk stratification in PAD.

α-Hydroxybutyrate dehydrogenase (HBDH) is a marker of cell death particularly reflecting renal, red blood cell and myocardial damage^10–14^. Since chronic kidney disease, anemia and myocardial injury predispose patients to adverse events following percutaneous coronary intervention^10,15–20^, we hypothesized that HBDH may be associated with ischemic outcomes following infrainguinal angioplasty and stenting for stable PAD.

## Methods

### Patients

In this prospective cohort study, 83 patients undergoing successful infrainguinal angioplasty with endovascular stent implantation were enrolled consecutively at the Division of Vascular Medicine of the Medical University of Vienna^21^. All patients had intermittent claudication classified as Rutherford stages of PAD 2–3 due to sonographically confirmed infrainguinal artery stenosis and occlusion, respectively. All patients received long-term aspirin therapy (100 mg/day), and 75 mg of clopidogrel per day for three months following angioplasty and stenting. Clinical follow-up was assessed 1 and 2 years after the percutaneous intervention.

Exclusion criteria were a known aspirin or clopidogrel intolerance (allergic reactions, gastrointestinal bleeding), a therapy with vitamin K antagonists (warfarin, phenprocoumon, acenocoumarol) or direct oral anticoagulants (dabigatran, rivaroxaban, apixaban and edoxaban), a treatment with ticlopidine, dipyridamol or nonsteroidal antiinflammatory drugs, a family or personal history of bleeding disorders, malignant paraproteinemias, myeloproliferative disorders or heparin-induced thrombocytopenia, severe hepatic failure, known qualitative defects in thrombocyte function, a major surgical procedure within one week before enrollment, a platelet count <100.000 or >450.000/µl and a haematocrit <30%^22^.

The study protocol was approved by the Ethics Committee of the Medical University of Vienna in accordance with the Declaration of Helsinki and written informed consent was obtained from all study participants.

### Blood sampling

As previously described^22^, blood was drawn by aseptic venipuncture from an antecubital vein using a 21-gauge butterfly needle (0.8 × 19 mm; Greiner Bio-One, Kremsmünster, Austria) one day after the percutaneous intervention. To avoid procedural deviations all blood samples were taken by the same physician applying a light tourniquet, which was immediately released and the samples were mixed adequately by gently inverting the tubes.

### Measurement of HBDH, lactate dehydrogenase (LDH) and free haemoglobin

HBDH, LDH and free haemoglobin were measured in the central laboratory of the Medical University of Vienna according to standardized protocols.

### Clinical endpoints

Clinical follow-up was assessed at regular visits of the study participants to the outpatient department of the Division of Vascular Medicine at the Medical University of Vienna and via telephone calls, respectively. The primary endpoint was defined as the composite of the first occurrence of nonfatal myocardial infarction, nonfatal stroke or transient ischemic attack, and cardiovascular death within 2 years after angioplasty and stenting^21^. Target vessel restenosis > 80% or reocclusion as assessed by duplex sonography was defined as secondary endpoint.

### Sample size calculation and statistical analysis

A sample size calculation was based on the observed mean ± SD (108 ± 26 U/L) of HBDH in a former population of 30 stable PAD patients (15 male, 15 female; age 64 years (58–71 years)) 24 hours after angioplasty and stenting^23–25^. We calculated that we needed to include 80 patients to be able to detect a 30% relative difference of HBDH between patients without and with the primary endpoint with a power of 83% (using a two-sided alpha level of 0.05). To compensate for loss to follow-up we included 3 additional patients^24^.

Statistical analysis was performed using the Statistical Package for Social Sciences (IBM SPSS version 25, Armonk, New York, USA). Median and interquartile range of continuous variables are shown. Categorical variables are given as number (%). We performed Mann Whitney U tests to detect differences of continuous variables. The chi-square test and the Fisher´s exact test were used to detect differences in categorical variables, respectively^24^. Receiver operating characteristic (ROC) curve analysis was used to determine the ability of HBDH to distinguish between patients without and with the primary endpoint^25^. The p-value was determined using the DeLong test. The optimal cut-off value was calculated by determining the HBDH level that provided the greatest sum of sensitivity and specificity. A survival curve was generated using the Kaplan-Meier method, and the difference between the groups was assessed by the log-rank test. Two-sided p-values < 0.05 were considered statistically significant^21,24,25^.

## Results

Characteristics of the overall study population and of patients without and with the primary endpoint are shown in Table 1. HBDH in the overall study population was 106 U/L (95–123 U/L). Of note, none of the included patients had suffered myocardial infarction within 6 months prior to infrainguinal angioplasty and stenting. Moreover, a history of myocardial infarction was present in 18.2% and 16.7% of patients without and with the primary endpoint, respectively (Table 1; p = 1). Twenty-eight (33.7%) and 23 (27.7%) patients of the study population had documented stable coronary artery disease (CAD) and cerebrovascular disease (CVD) at study inclusion, respectively. The presence of stable CAD and CVD at baseline did not differ significantly between patients without and with the primary endpoint (Table 1; both p ≥ 0.7). Polyvascular disease was documented in 41 patients (49.4%) at study inclusion (10 patients with PAD, CAD and CVD; 18 patients with PAD and CAD; 13 patients with PAD and CVD; Table 1). The presence of polyvascular disease at baseline did not differ significantly between patients without and with the primary endpoint (38 (49.4%) vs. 3 (50%) patients; p = 1).

**Table 1:** Clinical, laboratory and procedural characteristics of the overall study population, and of patients without and with the primary endpoint.

Characteristics	overall (n = 83)	no primary endpoint (n = 77)	primary endpoint (n = 6)	p
**Demographics**				
Age, years	65 (58–74)	64 (58–72)	73 (66–81)	0.2
Male sex, n (%)	51 (61.4)	48 (62.3)	3 (50)	0.7
Body mass index, kg/m^2^	26.8 (25.7–29)	26.7 (24.5–28.7)	27.1 (26.4–29)	0.6
**Medical history**				
Hypertension	78 (94)	72 (93.5)	6 (100)	1
Hypercholesterolemia	77 (92.8)	71 (92.2)	6 (100)	1
Diabetes mellitus	29 (34.9)	28 (36.4)	1 (16.7)	0.7
Active smoking	35 (42.2)	34 (44.2)	1 (16.7)	0.4
Previous myocardial infarction	15 (18.1)	14 (18.2)	1 (16.7)	1
CAD	28 (33.7)	26 (33.8)	2 (33.3)	1
CVD	23 (27.7)	21 (27.3)	2 (33.3)	0.7
PAD, CAD + CVD	10 (12)	9 (11.7)	1 (16.7)	0.6
PAD + CAD	18 (21.7)	17 (22.1)	1 (16.7)	1
PAD + CVD	13 (15.7)	12 (15.6)	1 (16.7)	1
**Laboratory data**				
Haemoglobin, g/dL	13.7 (12.5–14.7)	13.7 (12.6–14.7)	12.3 (11–14.6)	0.3
Haematocrit, %	40.2 (37–42.9)	40.4 (37.2–43.2)	35.9 (33.8–42.9)	0.2
White blood cell count, G/L	8.7 (6.8–10.5)	9 (6.8–10.5)	7.5 (6.7–8.4)	0.2
Platelet count, G/L	210 (181–237)	210 (181–230)	231 (207–249)	0.5
HBDH, U/L	106 (95–123)	105 (95–120)	126 (116–137)	0.04
Lactate dehydrogenase, U/L	167 (145–190)	167 (145–187)	179 (166–229)	0.2
Free haemoglobin, mg/dL	2.1 (1.4–3.3)	2.1 (1.4–3.4)	1.7 (1.3–2)	0.2
Serum creatinine, mg/dL	1 (0.9–1.2)	1 (0.9–1.1)	1.2 (1–1.4)	0.2
C-reactive protein, mg/dL	1.1 (0.4–1.8)	1.1 (0.4–1.8)	1.3 (0.4–4.3)	0.4
**Procedure**				
Stent implantation	83 (100)	77 (100)	6 (100)	1
Number of stents/patient	2 (1–2)	2 (1–2)	2 (1–2)	0.8
**Medication pre-intervention**				
Clopidogrel	83 (100)	77 (100)	6 (100)	1
Aspirin	83 (100)	77 (100)	6 (100)	1
Statins	74 (89.2)	68 (88.3)	6 (100)	1
ACE inhibitors/ARB	72 (86.7)	66 (85.7)	6 (100)	1
Beta blockers	51 (61.4)	47 (61)	4 (66.7)	1
Calcium channel blockers	34 (41)	31 (40.3)	3 (50)	0.7
Proton pump inhibitors	39 (47)	37 (48.1)	2 (33.3)	0.7

Continuous data are shown as median (interquartile range). Dichotomous data are shown as n (%).

ACE, angiotensin converting enzyme; ARB, angiotensin receptor blockers; CAD, coronary artery disease; CVD, cerebrovascular disease; HBDH, α-hydroxybutyrate dehydrogenase; PAD, peripheral artery disease.

The primary endpoint occurred in 6 patients (7.2% of the study population) within 2 years after peripheral angioplasty with stent implantation and comprised 2 nonfatal myocardial infarctions, 2 nonfatal strokes, 1 transient ischemic attack and 1 cardiovascular death. Target vessel restenosis or reocclusion occurred in 28 patients (33.7%) during follow-up.

**Figure 1:**
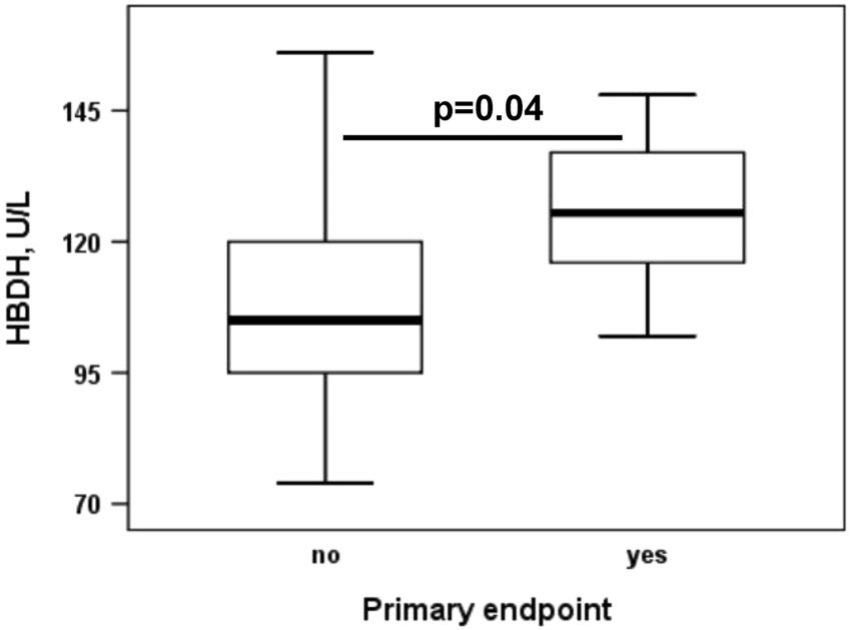
α-Hydroxybutyrate dehydrogenase (HBDH) in patients without and with the primary endpoint. The boundaries of the box show the lower and upper quartile of data, and the line inside the box represents the median. Whiskers are drawn from the edge of the box to the highest and lowest values that are outside the box but within 1.5 times the box length.

HBDH levels at baseline were significantly higher in patients who subsequently developed the primary endpoint (Fig. 1; Table 1; 126 U/L [116–137 U/L] vs. 105 U/L [95–120 U/L]; p = 0.04). In contrast, LDH and free haemoglobin levels did not differ significantly between patients without and with atherothrombotic events during follow-up (Table 1; LDH: 167 U/L [145–187 U/L] vs. 179 U/L [166–229 U/L]; free haemoglobin: 2.1 mg/dL [1.4–3.4 mg/dL] vs. 1.7 mg/dL [1.3–2 mg/dL]; both p ≥ 0.2). HBDH, LDH and free haemoglobin were similar in patients without and with target vessel restenosis or reocclusion within 2 years after angioplasty and stenting (all p > 0.6).

**Figure 2:**
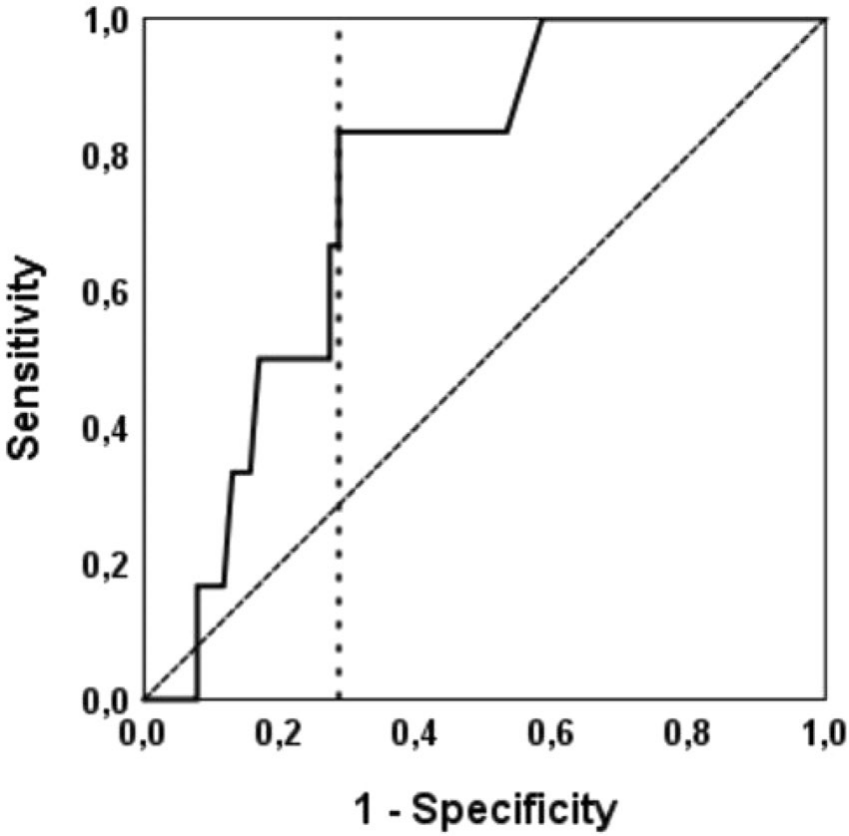
Receiver-operating characteristic curve for the analysis of the predictive value of α-hydroxybutyrate dehydrogenase (HBDH) for the primary endpoint. An area under the curve of 0.753 was observed (95% CI 0.61–0.9; p = 0.04). The cut-off value for high HBDH is shown as vertical dotted line originating from the x-axis.

**Figure 3:**
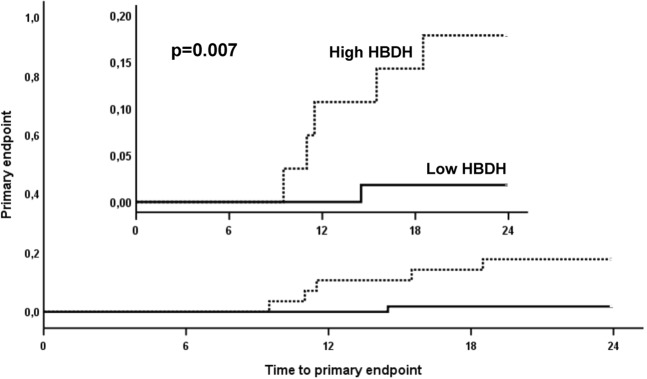
Kaplan-Meier analysis for the cumulative incidence of the primary endpoint in patients with high and low α-hydroxybutyrate dehydrogenase (HBDH). High HBDH is indicated by the dotted line, low HBDH is indicated by the solid line.

ROC curve analysis revealed that HBDH could distinguish between patients without and with future atherothrombotic events (Fig. 2; area under the curve = 0.753, 95% CI 0.61–0.9; p = 0.04). A HBDH concentration ≥ 115 U/L was identified as the best threshold to predict the composite endpoint, providing a sensitivity of 83.3% and a specificity of 71.4%, and was therefore defined as high HBDH. High HBDH was seen in 28 patients (33.7% of the study population). Ischemic events occurred significantly more often in patients with high HBDH than in patients with lower HBDH levels (Fig. 3; 5 vs. 1 patients, p = 0.007 with the log-rank test).

## Discussion

Our study is the first to investigate the association of markers of cell death with clinical outcomes following infrainguinal angioplasty and stenting for PAD. High levels of HBDH were linked to an increased risk of atherothrombotic events over 2 years, whereas target lesion restenosis and reocclusion did not occur more frequently in patients with high HBDH. Plasma concentrations of LDH and free haemoglobin were not linked to atherothrombotic events and target lesion restenosis or reocclusion, respectively.

HBDH was investigated as a potential risk marker because it reflects renal, red blood cell and myocardial injury^10–14^. In order to study whether cell death in general or red cell damage alone are linked to ischemic outcomes, we decided to also measure LDH and free haemoglobin concentrations, respectively. Our results, however, point towards HBDH as most promising risk predictor of atherothrombotic events among these 3 markers of cell injury. Of note, serum creatinine, haemoglobin and haematocrit alone did not differ significantly between patients without and with the primary endpoint in our study (Table 1). However, serum creatinine was numerically higher and both haemoglobin and haematocrit were numerically lower in patients who subsequently developed the primary endpoint. It may therefore be speculated that by combining information on subclinical kidney, red blood cell and myocardial injury, HBDH may represent a more sensitive risk marker for atherothrombotic outcomes than the respective laboratory values alone.

Considering the inclusion of patients with manifest atherosclerotic cardiovascular disease, we observed a rather low rate of atherothrombotic events during follow-up. This may be due to 1. the inclusion of stable PAD patients undergoing elective infrainguinal angioplasty and stenting because of intermittent claudication and 2. optimal medical treatment and risk factor management following the percutaneous intervention. In detail, all patients received state-of-the-art antiplatelet, antihypertensive and lipid-lowering therapy and had regular check-ups at the outpatient department every 6 months^2^. As a next step, it would be interesting to study the predictive value of HBDH for atherothrombotic outcomes in high-risk patients. Among PAD patients, especially those suffering critical limb ischemia may benefit from adequate risk stratification in order to receive an individually tailored treatment approach and follow-up strategy. Finally, HBDH may also be linked to the prognosis of patients with chronic or acute ischemic heart disease and cerebrovascular disease, respectively.

The lack of a significant association between HBDH levels and target vessel restenosis or reocclusion may be explained by differences in the underlying pathomechanisms: Target vessel restenosis usually occurs due to intimal hyperplasia potentially resulting from chronic inflammation^26^. In contrast, myocardial infarction, stroke and cardiovascular death primarily arise from a prothrombotic environment^6^. Our finding of an association between HBDH and atherothrombotic events suggests that HBDH levels might mirror the latter. However, large clinical trials confirming HBDH as predictor of thrombotic outcomes are needed before HBDH can be considered for risk stratification in PAD.

Due to the small sample size, our study should be considered hypothesis-generating only. Moreover, we exclusively enrolled stable PAD patients who underwent elective infrainguinal angioplasty and stenting due to intermittent claudication. Therefore, our results cannot be extrapolated to patients with critical limb ischemia. Elevated HBDH levels may be attributable to individual reactions to the peripheral interventions. Alternatively, HBDH might have already been increased before angioplasty and stenting in some patients, reflecting increased cell turnover or damage. Unfortunately, all markers of cell death were only determined 24 hours after the intervention. Consequently, we cannot provide preprocedural HBDH values or data on the variability of HBDH levels over time. This time point was chosen because (1) 24 hours after the elective procedures, all patients were still at the inpatient ward, and (2) we sought to investigate whether or not a single postprocedural HBDH measurement may be used for risk stratification. Further, the AUC of 0.753 observed in the ROC curve cannot be considered a strong classifier. Finally, we do not provide mechanistic data supporting the above-discussed speculations.

In conclusion, HBDH is associated with the occurrence of atherothrombotic events after infrainguinal angioplasty with stent implantation. Future trials are warranted to study the predictive role of HBDH for adverse outcomes in patients with critical limb ischemia and other manifestations of cardiovascular disease, and to investigate underlying mechanisms.

## Data Availability

The datasets generated during and analysed during the current study are available from the corresponding author on reasonable request.
